# Efficacy of Transurethral Resection of the Prostate in Male Patients With Impaired Detrusor Contractile Function and Urinary Retention

**DOI:** 10.1111/luts.70040

**Published:** 2025-11-11

**Authors:** Balázs Kenyeres, Alexandra Helmeczi, Ákos Pytel

**Affiliations:** ^1^ Department of Urology University of Pécs Pécs Hungary; ^2^ Medical School University of Pécs Pécs Hungary

**Keywords:** detrusor underactivity, TURP, urinary retention

## Abstract

**Objectives:**

Detrusor underactivity (DUA) increasingly affects aging male patients with voiding symptoms, while its management remains challenging, with less favorable surgical outcomes compared to bladder outlet obstruction. Our aim was to evaluate the efficacy of TURP in male patients with urinary retention and unfavorable urodynamic findings.

**Materials and Methods:**

This retrospective, single‐center study included 67 male patients undergoing TURP between September 2021 and September 2024 after a failed trial of voiding. Patients were divided into three groups labeled as detrusor acontractility (DA, *n* = 18, voided without detrusor contraction), DUA (*n* = 19, voided with BCI < 100 and BOOI < 20), or non‐voiders (*n* = 30, failed to urinate and lacked measurable detrusor contractions on pressure‐flow study). Surgical success was defined as successful voiding with post‐void residual (PVR) < 150 mL at 3 months. Baseline parameters (PSA, prostate volume, cystoscopy and urodynamic findings), rate of surgical success, Patient Global Impression of Improvement (PGI‐I) score and adverse events (subsequent surgeries and urinary tract infection) were registered and analyzed.

**Results:**

Overall 37 (55.2%) patients became catheter‐free within 3 months. The mean follow‐up duration was 25.4 ± 9.6 months. Surgical success was achieved in DA, DUA, and non‐voider groups in 6 (33%), 13 (68.4%), and 18 (60%) cases, respectively, and a PGI‐I score greater than 4 was reported by 35 (52.2%) patients. Multivariate analysis showed higher prostate volume as an independent predictor for failure (OR: 1.7; 95% CI: 1.010–2.940; *p* = 0.046). Two patients developed stress urinary incontinence, and three required additional surgical intervention due to urethral stricture. Urinary tract infections occurred more frequently in the treatment failure group: Nine patients (30%) were hospitalized, and 16 (53%) required more than two antibiotic prescriptions within a 6‐month period. In contrast, among the success group, only two patients (5.4%) were hospitalized, and none required frequent antibiotic therapy.

**Conclusion:**

TURP offers a reasonable chance for catheter discontinuation in case of unfavorable urodynamic parameters. With careful patient selection in mind, surgery remains a viable option even in this patient population.

## 1 Introduction

Lower urinary tract symptoms (LUTS) are highly prevalent in the male population, particularly with advancing age. According to a recent global meta‐analysis, 63.2% of men experience some form of LUTS, with voiding symptoms affecting 36% of this population. Moderate and severe symptom burdens were reported in 24% and 6% of cases, respectively [1]. Acute urinary retention (AUR) represents one of the most severe clinical manifestations of voiding dysfunction, with an incidence of 2.8–6.8 per 1000 man‐years in the general population and up to 18.3 per 1000 man‐years in men affected by LUTS [2, 3].

AUR results from bladder outlet obstruction (BOO), detrusor underactivity (DUA) or a combination of both. The initial management of LUTS and AUR focuses on conservative treatment; however, performing surgical intervention aimed at reducing bladder outlet resistance is a common choice in refractory cases. The gold standard for characterizing underlying voiding dysfunction is the pressure‐flow urodynamic study (PFS), which helps differentiate BOO from DUA. BOO is more precisely defined and better understood than DUA. The International Continence Society (ICS) characterizes BOO as reduced urine flow with simultaneously elevated detrusor pressure during voiding. A widely used parameter for assessing obstruction is the Bladder Outlet Obstruction Index (BOOI). Patients with values above 20 are considered to have some degree of obstruction [4].

In contrast, DUA remains less well defined. The ICS defines it as low detrusor pressure or short contraction duration, typically combined with reduced flow rate, resulting in prolonged or incomplete bladder emptying [4]. The most commonly used parameter to assess detrusor contractility is the Bladder Contractility Index (BCI), which estimates the projected isovolumetric pressure (PIP), using empirically defined slopes and boundaries on the pressure–flow plot [5]. Values below 100 are generally accepted to reflect impaired contractility.

Patients with high BOOI and preserved contractility (BCI > 100) are considered ideal candidates for benign prostatic hyperplasia (BPH) surgery. However, outcomes in patients with low BOOI or impaired contractility are less predictable [6, 7].

The prevalence of DUA in the general male population remains uncertain, primarily due to overlapping symptomatology with BOO. In patients undergoing urodynamic evaluation for LUTS, DUA prevalence ranges from 23%–25% [8, 9]. In retrospectively analyzed cohorts undergoing transurethral resection of prostate (TURP), the rate of preoperative catheter dependence was as high as 48% in patients with either pure DUA or mixed presentation with BOO [10].

In patients with urinary retention and low BOOI, the role of surgery remains controversial. In such cases, long‐term management often involves clean intermittent catheterization or in selected cases sacral neuromodulation. Evidence suggests that failure rates for TURP are significantly higher in men with unfavorable urodynamic findings, particularly those with acontractile or hypocontractile detrusor [11].

The aim of our study was to evaluate the efficacy of TURP in male patients with a failed trial of voiding, requiring indwelling catheterization (IDC) or clean intermittent self‐catheterization (CISC), who presented with unfavorable urodynamic parameters predictive of poor surgical outcomes.

## 2 Materials and Methods

Our retrospective, single‐center study was conducted at a university hospital's urodynamic unit, involving patients urodynamically evaluated and surgically treated between September 1, 2021 and September 1, 2024. The study was approved by the regional ethics committee (approval number: KK\517‐1\2025).

Inclusion criteria were male patients over 18 years of age, who underwent TURP after a failed trial of voiding requiring IDC or CISC and had preoperative PFS indicating impaired detrusor function. Patients were categorized into three groups according to the findings of the voiding phase of PFS. Patients were divided into the DA group if the absence of measurable detrusor contraction during voiding was observed. They were categorized into the DUA group, if during voiding BCI < 100 and BOOI < 20 were observed. Patients who were unable to void during PFS and exhibited no measurable detrusor contractions were allocated to the non‐voider group. Patients were excluded if there was no trace of a minimum of 3‐month postoperative follow‐up. Other exclusion criteria included neurogenic bladder, prior urethral stricture, previous TURP and previous or ongoing oncologic treatment for prostate cancer.

**FIGURE 1:**
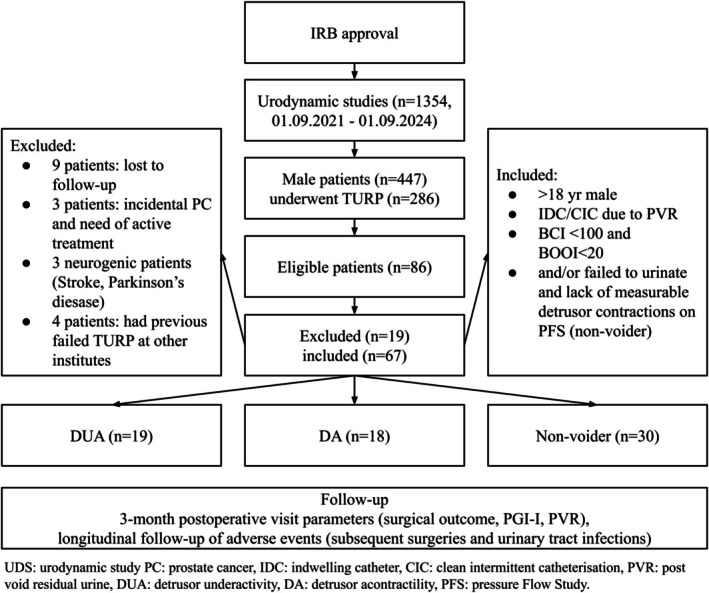
Study flow and patient selection criteria.

A total of 1354 urodynamic studies were identified during the study period, of which 447 involved male patients. Among them, 286 underwent TURP. Based on the inclusion criteria, 86 patients were deemed potentially eligible. Nineteen patients were excluded because they were either lost to follow‐up before the 3‐month visit; had a diagnosis of incidental prostate cancer requiring active treatment; had a history of neurogenic disease (stroke, Parkinson's disease and incomplete spinal cord lesion); or had previously failed TURP procedures performed at other institutions. Finally, 67 patients met all inclusion criteria and were included in the final analysis. The study flow is summarized in Figure 1.

All urodynamic procedures were performed according to ICS standards [12]. Wiest Sirius 8000 system (Wiest Medizintechnik, Germany) and 8 Ch multichannel water‐filled catheters for measuring vesical pressure were used. BCI and BOOI were calculated using ICS formulas where BCI = 5 × QmaxPFS + PdetQmax, and BOOI = PdetQmax−2 × QmaxPFS. A post‐void residual volume (PVR) > 150 mL was the threshold for IDC or CISC initiation.

The following preoperative parameters were collected and analyzed: prostate volume (mL); serum prostate‐specific antigen (PSA, ng/mL); PVR (mL); proportion of patients with detrusor overactivity (DO); urethrocystoscopic signs of bladder wall trabeculation or signs of BOO. BOO was considered present when narrowing of the posterior urethra due to enlarged lateral prostatic lobes or a constricted bladder neck was observed.

Postoperative evaluation results were collected from patient visit records at the 3‐month mark. Surgical success was defined as the cessation of catheter usage (IDC/CISC) and a postoperative PVR of less than 150 mL at 3 months, maintained throughout the study period. The PVR threshold of 150 mL was arbitrarily selected, which is commonly used in clinical settings, reflects our own clinical practice and is at least supported by existing literature [13]. Patient‐reported satisfaction was assessed using the Patient Global Impression of Improvement (PGI‐I) scale. A PGI‐I score greater than 4 was interpreted as a clinically meaningful improvement. Adverse events, including the need for additional surgical interventions and suspected urinary tract infections (UTI), were monitored throughout the study. UTI‐related outcomes included hospitalization in the early (< 3 months) and late (> 3 months) postoperative periods, as well as antibiotic (AB) prescriptions for UTI indications.

Continuous variables are reported as means ± standard deviations. Distribution normality was assessed using the Shapiro–Wilk test. For comparisons between groups, the two‐sample *t*‐test was used for normally distributed data; otherwise the Mann–Whitney U‐test was applied. Associations between categorical variables were assessed using the Chi‐squared test. To identify independent risk factors for surgical failure, logistic regression analysis with possible predictors was performed. All analyses were conducted using SPSS Statistics software version 26 (IBM Corp., Armonk, NY, USA). A *p*‐value < 0.05 was considered statistically significant.

## 3 Results

Baseline characteristics of the patients are detailed in Table 1. The mean age at the time of TURP was 74 ± 11.1 years, with an average follow‐up duration of 25.4 ± 9.6 months. Eighteen (26.9%) patients were diagnosed with DA, 19 (28.3%) with DUA and 30 (44.8%) were allocated to the non‐voider group. Detrusor overactivity (DO) was identified in 20 cases (29.6%) during preoperative urodynamic evaluation. Cystoscopic assessment described bladder wall trabeculation in 57 patients (81%) and signs of BOO in 61 patients (91%).

**TABLE 1:** Baseline patient characteristics.

	Total	DA	DUA	Non‐voiders
*n*	67	18	19	30
Age	74 ± 11.1	73.3 ± 1	78.3 ± 12.2	71.6 ± 15.5
PRvol (mL)	60.9 ± 24.1	62.8 ± 3.6	61.3 ± 28.4	59.5 ± 36
PSA (ng/mL)	2.5 ± 2.7	1 ± 0.7	2.5 ± 1	3.5 ± 3.6
PVR (mL)	455 ± 292	360 ± 187	426 ± 195	531 ± 371
BOOI		−6 ± 2.9	2.7 ± 4.4	—
BCI		15 ± 7.3	44.7 ± 15.1	—
cysObstruction	61 (91)	12 (67)	19 (100)	30 (100)
cysTrabeculation	54 (81)	12 (67)	12 (63)	30 (100)

Abbreviations: BCI, bladder contractility index; BOOI, bladder outlet obstruction index; cysObstruction, BOO signs on cystoscopy; cysTrabeculation, sign of bladder wall trabeculation on cystoscopy; DA, detrusor acontractility; DUA, detrusor underactivity; Non‐voiders, unable to void during PFS and exhibiting no measurable detrusor contractions; PRvol, prostate volume; PVR: Post Void Residual urine (mL).

At the 3‐month postoperative evaluation, 37 (55.2%) patients met the treatment success criteria and voided with significantly lower PVR (14.2 ± 14.3 mL vs. 547 ± 331 mL, *p* < 0.001). Variable characteristics observed in the treatment success and failure groups are summarized in Table 2. Surgical success was achieved in 6 (33%), 13 (68.4%), and 18 (60%) patients from DA, DUA and non‐voider groups, respectively, highlighting a markedly poorer outcome in DA patients. Seventeen of the 35 patients (52.2%) reported > 4 subjective improvements on the PGI‐I scale; all of them belonged to the surgical success group (*p* < 0.001). Two patients reported dissatisfaction due to postoperative stress urinary incontinence; one of them had preoperative DO.

**TABLE 2:** Variable characteristics in success and failure groups.

	Success	Failure
	*n* = 37 (55.2%)	*n* = 30 (44.8%)
Age (years)	70.3 ± 13.4	78.4 ± 5.8
Prostate volume (mL)	48.1 ± 15.3	76.3 ± 25
PSA (ng/mL)	1.5 ± 1.3	3.8 ± 3.4
Post‐void residual (mL)	314.4 ± 51.4	629 ± 366
Detrusor acontractility	6 (33)	12 (67)
Detrusor underactivity	13 (68.4)	6 (31.6)
Non‐voider	18 (60)	12 (40)
Cystoscopic obstruction	31 (84)	30 (100)
Cystoscopic trabeculation	24 (65)	30 (100)
UTI hospitalization < 3 months	1 (2.7)	0 (0)
UTI hospitalization > 3 months	2 (5.4)	9 (30)
> 2 AB courses/6 months	0 (0)	16 (53)

Abbreviations: AB/6 months, number of average antibiotic therapy courses required every 6 months due to UTI; Cystoscopic obstruction, cystoscopic signs of bladder otlet obstruction; Cystoscopic trabeculation, cystoscopic signs of bladder wall trabeculation; DA, detrusor acontractility; DUA, detrusor underactivity; Non‐voider, unable to void during PFS and exhibiting no measurable detrusor contractions; PRvol, prostate volume; PVR, preoperative post‐void residual; Success, catheter‐free after surgery; UTI hospitalization, number of patients hospitalized before or after the 3‐months postoperative visit due to UTI.

Univariate analysis revealed significant associations between treatment failure and the following parameters: older age, higher PSA, greater prostate volume, higher preoperative PVR and DA. Multivariate analysis using continuous variables identified only higher prostate volume as a significant independent predictor of treatment failure (OR: 1.7; 95% CI: 1.010–2.940; *p* = 0.046).

Neither cystoscopic trabeculation (*p* = 0.998) nor BOO signs (*p* = 0.999), were predictive of surgical outcome, based on our evaluation criteria. The results of the uni‐ and multivariate analyses are presented in Table 3.

**TABLE 3:** Logistic regression analysis of preoperative risk factors.

Univariate logistic regression analysis				Multivariate logistic regression analysis of continuous variables			
Variable	OR	95% CI	*p*	Variable	OR	95% CI	*p*
Age (years)	1.089	1.023–1.159	0.008*	Age (years)	0.997	0.408–2.434	0.994
PRvol (mL)	1.430	1.155–1.770	0.001*	PRvol (ml)	1.723	1.010–2.940	0.046*
PSA (ng/mL)	1.897	1.221–2.948	0.004*	PSA (ng/ml)	1.796	0.80–40.449	0.713
PVR (mL)	1.009	1.004–1.014	< 0.001*	PVR (ml)	1.008	0.955–1.021	0.206
DA	3.157	0.998–9.990	0.05*				
DUA	0.482	0.157–1482	0.203				
Non‐voider	1.342	0.503–3.578	0.556				
CysObstruction	1.51 × 10^9^	0.00 – undef.	0.999				
CysTrabeculation	1.95 × 10^9^	0.00—undef.	0.998				

*Note:* An asterisk (*) indicates statistical significance at the *p* < 0.05 level.

Abbreviations: CI, confidence interval; cysObstruction, BOO signs on cystoscopy; cysTrabeculation, sign of bladder wall trabeculation on cystoscopy; DA, detrusor acontractility; DUA, detrusor underactivity; Non‐voider, failed to urinate and lack of measurable detrusor contractions on pressure‐flow study; OR, odds ratio for treatment failure; PRvol, prostate volume (mL); PVR, preoperative post void residual urine volume (mL).

Two patients died during the follow‐up period due to causes unrelated to surgery or perioperative hospitalization (heart failure and pneumonia). Three patients required subsequent surgical intervention during follow‐up owing to urethral stricture formation.

One patient (1.5%) required early hospitalization for UTI postoperatively. During follow‐up, UTI incidence was lower in the treatment success group: 2 patients (5.4%) were hospitalized, 18 (49%) received AB treatment, and none received more than two AB courses (mean 0.21 ± 0.29 prescriptions over 6 months). By contrast, in the failure group, 9 patients (30%) were hospitalized, all received UTI treatment, and 16 (53%) required more than two AB courses (mean 1.99 ± 1.26 prescriptions over 6 months).

## 4 Discussion

The evaluation of voiding function traditionally relies on parameters such as detrusor pressure and urine flow to estimate detrusor contraction strength and outflow resistance. Considerable debate persists regarding the optimal management of patients with impaired detrusor function, particularly concerning patient cohorts who may not derive substantial benefit from surgical intervention.

Long‐term cohort studies question sustained benefits of TURP in DUA. Thomas et al. found that TURP yielded minimal, non‐significant long‐term urodynamic or symptomatic improvements in DUA patients; on the other hand, those on CISC maintained stable urodynamic parameters, while avoiding surgical risks [11]. Similarly, Al‐Hayek et al. concluded that detrusor contractility neither deteriorates with chronic BOO nor improves post‐TURP, suggesting DUA typically persists without progression [14].

Regarding the safety of surgical intervention, a systematic review and meta‐analysis by Wroclawski et al. suggests that surgery in DUA patients is at least as safe as in those with normal detrusor contractility and BOO [15]. However, when the primary therapeutic goal is the improvement in symptoms and urodynamic outcomes, the success rate appears less favorable in DUA patients compared to those with good contractility. Chuang et al. investigated surgical outcomes in DUA patients and found that a lower BOOI correlated with a poorer success rate of achieving at least a 50% reduction in IPSS in 76%, 64%, and 27% for BOOI > 40, 20–40, and < 20, respectively [10]. Likewise, Zhu et al. based on similar criteria reported inferior success rates in the case of low BCI and BOOI parameters (38%–41%) compared to patients with obstruction and good contractility, where success exceeded 80% [6]. Conversely, Lebani et al. presented contrasting findings comparing groups of BOO, BOO + DUA and DUA patients. While they observed significantly less improvement in urine flow parameters disfavoring pure DUA patients post‐surgery (4.6 mL/s vs. 8.6–9 mL/s), the subjective success measured by IPSS changed from baseline 24.5–24.9 to 4.6–6.6 points, resulting in nearly identical improvement across all three groups [7]. It is noteworthy that the latter patient group averaged higher IPSS baseline values compared to the cohort of Thomas et al. (24 vs. 13), which may explain the conflicting conclusions [11].

A different perspective also emerges when the primary aim is catheter discontinuation. Sagen et al. [16] reported high decatheterization rates with TURP (83%) in general BPH patients, with unknown invasive urodynamic parameters, which trend appears to extend to elderly populations as well [17]. The findings by Chuang et al. also support this observation, indicating that while lower BOOI in DUA patients was associated with a less favorable subjective success rate (IPSS reduction), similarly to our results, the rate of decatheterization after surgery was notably high [10]. Revising the data of Thomas et al. from this perspective, discrepancies can be seen between their patient population and our cohort [11]. Their pivotal study involved patients with higher BCI (70 vs. 45), BOOI values (15 vs. 2.7) and lower PVR (100 mL vs. 460 mL) with a lower proportion requiring catheterization. It is challenging to ascertain the exact number of urinary retention resolutions from their publication. Of 52 patients under watchful waiting, eight eventually underwent TURP due to symptoms or retention progression, with only two requiring long‐term catheterization. It is plausible that the DUA patients in their study had less decompensated voiding, such that the reduction in outflow resistance achievable with TURP did not translate into dramatic subjective or urodynamically measurable changes. In patients with more severely decompensated voiding (e.g., requiring catheter), even a modest improvement in outflow may lead to a proportionally more significant recompensation and thereby a more substantial improvement in quality of life.

In the presence of DA, surgical success seems less favorable compared to DUA. Blaivas et al. found that following BOO surgery in the case of DA, 69% of patients remained on CISC and the subjective success rate was as low as 26% [18]. Conversely, in their DUA patient group a high subjective success rate of 98% was achieved. Preoperatively, 32% of these DUA patients were performing CISC, all of whom became catheter‐free post‐surgery. Comparing these findings to our data, we also observed a lower success rate in DA cases compared to both DUA and patients unable to urinate during PFS (33% vs. 60%–68.4%). Additionally, our DUA patients exhibited less favorable baseline BCI and BOOI values (44.7 vs. 54 and 2.7 vs. 23, respectively), which may account for our more modest outcomes compared to those reported by Blaivas et al.

Notably, satisfactory voiding can be achieved even without detrusor function at all. Patients with orthotopic neobladder (ONB) following radical cystectomy void using the Valsalva maneuver. Despite the lack of detrusor function in such patients, only 7.4% of them required catheterization, while 90% maintained good daytime continence, suggesting spared external urinary sphincter function [19]. Zhang et al. reported a mean maximal flow rate of 16.2 mL/s in ONB patients, an outcome considered favorable even for BPH/LUTS patients after TURP [20]. This demonstrates that well‐compensated voiding is feasible despite absent detrusor contractility. For a subset of ONB patients (5.4% of 15%) experiencing emptying problems, dysfunctional voiding was suggested as the cause, possibly due to a failure to synchronize sphincter relaxation with abdominal straining [21]. Such a mechanism likely affects the general population as well and highlights the frequently neglected role of the external urinary sphincter in voiding.

Regarding DUA patients with similarly unfavorable profiles, such as our cohort (low BCI, BOOI, and high PVR), Lee et al. reported a frequent (80%) improvement in symptoms and retention after TURP. The authors suggested the therapeutic success stemmed not only from obstruction relief but also from the disinhibition of the detrusor, potentially caused by hyper alpha‐adrenergic stimulation from the bladder neck area. Postoperatively, 69.4% of patients in the therapeutic success group exhibited improved detrusor function, with an increase in BCI (16.4 ± 19.8 to 61 ± 49.9) through a simultaneous rise in Qmax and PdetQmax, indicative of enhanced detrusor contraction strength [22]. Similar findings suggest return of contractility following prostate enucleation in men initially deemed acontractile by Mitchell et al. They reported good therapeutic efficacy in patients considered hypocontractile or acontractile with a decatheterization rate of over 94.7%. Notably, 78.9% of acontractile patients demonstrated newly apparent detrusor contractions during postoperative urodynamic studies [23]. Similar phenomena of detrusor function recovery have been observed following botulinum toxin A (BoNT‐A) usage in various forms of urethral sphincter dysfunction in non‐neurogenic populations, primarily documented by Kuo and colleagues. Besides its direct effect on muscle relaxation, BoNT‐A injections at the external urinary sphincter or bladder neck may lead to recovery of detrusor contractility, through chemical denervation and deactivation of afferent signals inhibiting the bladder reflex [24]. Following sphincter BoNT‐A injection, Kuo reported increased PdetQmax (7.4 ± 9.2 to 24 ± 13.1 cmH_2_O) and Qmax (4.7 ± 5.6 to 13 ± 6.6 mL/s) parameters from baseline, suggesting improved detrusor contractions [25]. However, these observations of potential detrusor function recovery must be juxtaposed with the long‐term follow‐up by Al‐Hayek et al., which did not support a lasting improvement in detrusor function after TURP [14].

Overall, growing evidence suggests that procedures aiming to improve voiding function through complex mechanisms extending beyond merely reducing outflow resistance. Dysfunctions of the bladder neck and external urethral sphincter may also alter voiding, either by contributing to outflow resistance themselves or by exerting an inhibitory effect on the detrusor muscle, even in neurologically intact individuals. The severity of DUA appears to have a differential impact on therapeutic success, depending on the specific treatment goals. Surgery in patients with DUA but compensated voiding (i.e., absence of high PVR) is perhaps less likely to yield unequivocally dramatic therapeutic outcomes in terms of urodynamic parameter changes. Conversely, for patients with significantly decompensated voiding and high PVR, despite unfavorable urodynamic characteristics, surgery may result in a greater impact on PVR reduction and quality of life. For this latter group, such surgery can exert a relatively more potent recompensating effect on voiding, potentially engaging all the aforementioned mechanisms.

A key strength of our study is its focus on a diagnostically challenging population for urologists performing urodynamics: patients lacking demonstrable detrusor contractility (e.g., non‐voiders), those with DA, or with low BCI and low BOOI indices. These three subgroups were analyzed separately, yielding notable insights.

Patients with greater prostate volumes and confirmed DA showed poorer outcomes. Importantly, non‐voiders without measurable detrusor contractions during PFS should not be hastily classified as DA; our findings suggest they more closely resemble the DUA phenotype. In the other two groups, over half of the patients regained spontaneous voiding, which was sustained over a mean follow‐up of 2 years. Regarding subsequent surgeries and early urinary tract infection (UTI) adverse events, we observed frequencies comparable to those reported in the literature for TURP in general patient populations [26, 27]. Data on UTIs following TURP, excluding early postoperative cases, remain limited. One cohort study reported that at least 20% of patients experience a UTI within 3 years post‐TURP [28]. In our older and clinically unfavorable cohort, this incidence was higher; however, the treatment success group showed a tolerable UTI burden. In contrast, the failure group exhibited increased rates of hospitalization and antibiotic use. Due to the absence of a representative control group, it remains unclear whether the elevated UTI burden in the failure group was a consequence of the unsuccessful surgery or pre‐existing factors such as long‐term indwelling catheter use. Nevertheless, this consideration and its potential risks should be discussed during patient consultation.

This study is limited by its small sample size and retrospective design, which reduce the statistical power and generalizability of our conclusions. Furthermore, the absence of postoperative urodynamic assessment and validated measures of symptom relief and quality of life hinders a more accurate evaluation of overall treatment success.

In conclusion, even in patients with unfavorable urodynamic findings, TURP offers a meaningful chance for catheter removal. Increasing evidence supports personalized decision‐making, helping clinicians weigh potential benefits against risks and better assess patient expectations and likely outcomes.

## Ethics Statement

This study was conducted in accordance with the ethical standards of the Declaration of Helsinki and was approved by the Regional Research Ethics Committee of the University of Pécs (KK\517‐1\2025).

## Conflicts of Interest

The authors declare no conflicts of interest.

## Data Availability

The data that support the findings of this study are available from the corresponding author upon reasonable request.
